# Varying the expression pattern of the strigolactone receptor gene *DAD2* results in phenotypes distinct from both wild type and knockout mutants

**DOI:** 10.3389/fpls.2023.1277617

**Published:** 2023-10-11

**Authors:** Revel S. M. Drummond, Hui Wen Lee, Zhiwei Luo, Jack F. Dakin, Bart J. Janssen, Kimberley C. Snowden

**Affiliations:** The New Zealand Institute for Plant and Food Research Limited, Auckland, New Zealand

**Keywords:** strigolactone, branch, development, gene expression, mutation, receptor, plant, petunia

## Abstract

The action of the petunia strigolactone (SL) hormone receptor DAD2 is dependent not only on its interaction with the PhMAX2A and PhD53A proteins, but also on its expression patterns within the plant. Previously, in a yeast-2-hybrid system, we showed that a series of a single and double amino acid mutants of DAD2 had altered interactions with these binding partners. In this study, we tested the mutants in two plant systems, *Arabidopsis* and petunia. Testing in *Arabidopsis* was enabled by creating a CRISPR-Cas9 knockout mutant of the *Arabidopsis* strigolactone receptor (AtD14). We produced SL receptor activity in both systems using wild type and mutant genes; however, the mutants had functions largely indistinguishable from those of the wild type. The expression of the wild type DAD2 from the CaMV 35S promoter in *dad2* petunia produced plants neither quite like the *dad2* mutant nor the V26 wild type. These plants had greater height and leaf size although branch number and the plant shape remained more like those of the mutant. These traits may be valuable in the context of a restricted area growing system such as controlled environment agriculture.

## 1 Introduction

The developmental trajectory of plants in response to genetic and environmental signals is complex. Plants integrate inputs that are heterogeneous in both time and space, requiring complex systems to sense and respond to those inputs. In large part, these programs are mediated and coordinated across the plant by small mobile signals (hormones) to regulate the output phenotypes. An evolutionarily conserved class of compounds, collectively known as strigolactones (SLs), mediate some of these input-to-output relationships in many if not all land plants, and are well characterized in rice, petunia, pea and *Arabidopsis* (Gomez-Roldan et al., 2008; Umehara et al., 2008; Waters et al., 2017). Both the SL pathway genes’ expression and the encoded proteins’ function are important to the full functionality of the signaling system: altering either or both might be expected to change the way the plants develop and respond to the environment. Thus, modifying either the expression or the function of the SL receptor might provide an avenue for generation of novel phenotypes.

The gene encoding the plant hormone receptor for SLs is expressed in a distinct and regulated pattern within plants (Arite et al., 2009). This pattern is controlled at both the transcriptional and post-transcriptional levels as part of an integrated system to balance root and shoot growth in response to developmental and environmental signals. Investigations in petunia (*Petunia x hybrida*) showed that transcript abundance of the receptor gene *DAD2* was altered depending on the plant organ, organ fate, phosphate availability and light quality (Drummond et al., 2015). Recent research in rice showed that the orthologous receptor gene *D14* is also controlled post-transcriptionally via the regulation of intron splicing in response to environmental signals (Liu et al., 2022). However, in *Arabidopsis* the expression patterns of the *AtD14* gene are largely unchanged by environmental inputs, with the *D14* binding partner *MAX2* appearing to be more dynamically regulated (Chevalier et al., 2014).

Ligand-protein and protein-protein interactions are core to signal reception and hence developmental regulation via strigolactones. The strigolactone signal transduction cascade is triggered by the ligand-induced formation of a protein complex containing the receptor protein (DAD2), a leucine-rich repeat F-Box protein (MAX2) and a large target protein involved in transcriptional repression via EAR domain motifs (D53) (Jiang et al., 2013; Zhou et al., 2013). In earlier work we generated several mutants in petunia DAD2 that allowed protein-protein interactions between the proteins of the strigolactone reception complex in the absence of the ligand (Lee et al., 2020). In particular, using a yeast-2-hybrid assay system we showed that the N242I mutant of DAD2 was able to interact with the petunia MAX2 protein (PhMAX2A) and that the F135A mutant of DAD2 was able to interact with the petunia D53 protein (PhD53A) in the absence of a ligand. These changes were independent, such that the N242I mutation did not allow interaction with PhD53A nor F135A with PhMAX2A. This suggests that the DAD2 protein interacts directly with both partners using different interaction surfaces. Additionally, we found that the three-way interaction of DAD2-PhMAX2A-PhD53A was also strigolactone independent when DAD2 had both the N242I and F135A mutations (Lee et al., 2020). These mutations to the strigolactone receptor have also been examined in a plant leaf transient assay system. Using *Arabidopsis* orthologues of the genes (AtD14 for DAD2 and SMXL7 for D53), White et al. (2022) showed that the protein interactions were more sensitive to strigolactone but were not strigolactone-independent for either the equivalent N242I mutant or the double mutant, with no change seen for the F135A mutant.

Petunia is a useful model system for studying plant development, and three mutants were identified in the mid-1990s that had reduced height, increased branching and delayed senescence (Napoli and Ruehle, 1996). Subsequent research identified the causative mutations for each of these (Snowden et al., 2005; Drummond et al., 2009; Hamiaux et al., 2012). The first two (*dad1* and *dad3*) were in the biosynthetic pathway for the strigolactone ligand and the third in the receptor (*dad2*). The original mutation of the *DAD2* gene was an insertion of a transposon, leading to a non-transgenic plant with a complete knockout of the gene. Since that time two additional mutant alleles have been recovered from the progeny of that plant, including *dad2-3*, a stable transposon-free allele, which was spontaneously formed when the transposon excised, in which the second exon was deleted. The *dad2* mutant of petunia could be complemented to a near wild type phenotype by the addition of a coding sequence copy (intron and UTRs removed) of the *DAD2* gene expressed from the CaMV 35S promoter (Hamiaux et al., 2012).

*Arabidopsis* is another model plant system used to great effect for testing the effects of mutations and of transgenes: most of the key genes in the biosynthetic and signal transduction pathways for strigolactones have well-characterized mutants (reviewed in Luo et al., 2021). With regard to the strigolactone receptor in *Arabidopsis* (*AtD14*), there are several published and well-characterized mutants (Waters et al., 2012; Chevalier et al., 2014). The most commonly used are a T-DNA insertion mutant (*Atd14-1*) and an EMS-generated point mutation (*d14-seto*). Although the first of these is a complete knockout of the gene, owing to its transgenic nature, it requires use of non-standard selection when used in further transformations and experimentation. The *d14-seto* mutation produces a plant with a phenotype similar but not identical to that of the *Atd14-1* mutant – as a full-length transcript and protein are made from the gene, there is a suggestion this does not lead to a loss-of-function allele (Li et al., 2022).

Here we present a series of experiments where we investigated the effects of the putatively strigolactone-independent mutants of DAD2, first in *Arabidopsis* and then in petunia. Although the yeast system was useful to rapidly screen a large number of mutant genes, we wanted to investigate the mutants in a plant system to determine whether the effect was observable in the native biological system. We include a new CRISPR-Cas9-mediated mutant of AtD14 (*Atd14-7*), which is both a complete knockout and non-transgenic; evidence that recognition of the intron in the petunia DAD2 gene is encoded within the coding sequence, and that this is inappropriately recognized by the spliceosome in *Arabidopsis*; and also that ectopically expressing the *DAD2* gene in petunia from a strong constitutive promoter fails to phenocopy the native gene with respect to both leaf shape and size and branch number.

## 2 Materials and methods

### 2.1 Genetic stocks and plant growth conditions

The wild type *Arabidopsis thaliana* used in these experiments was the Col-0 ecotype. The previously described mutants used in this work were *d14-1*, *d14-seto* and *max4-2*, all in a Col-0 background (Sorefan et al., 2003; Waters et al., 2012; Chevalier et al., 2014). Plants were grown in a growth room lit with LX601C LED lights (Heliospectra AB) with 16-h light: 8-h dark cycles in constant temperature 25 ± 2°C. In the greenhouse all plants were grown in commercial potting medium (potting and seed raising mix, Yates^®^, New Zealand). *Arabidopsis* was transformed as described by Clough and Bent (1998), with minor modifications in that the *Agrobacterium* cultures were applied in 50 mL of 5% sucrose and 0.02% Silwet^®^-77 in water and the rosette leaves were kept dry. Screening for transgenic seeds/plants was by culture on ½ MS (Murashige and Skoog, 1962) plates with 1% sucrose 0.8% agar and 100 µg/mL kanamycin. Prior to culture the seeds were surface-sterilized by incubation in 70% ethanol and 0.05% Triton™ X-100 for 10 minutes before washing in 100% ethanol and drying.

The wild type petunia used in these experiments was *Petunia hybrida* Vilm inbred line V26. The *dad1* and *dad2* mutants, derived from this line, were isolated by Napoli and Ruehle (1996). The particular allele of *dad2* (*dad2-2*) used in this work was a stable transposon footprint allele identified in Hamiaux et al. (2012). All experiments were conducted in greenhouse conditions under natural light (supplemented to maintain 16-h light periods, LX601C lamps, Heliospectra) and with temperature set at 25 ± 5°C. Plants were grown in commercial potting medium (potting and seed raising mix, Yates) watered daily with fertilizer 80 mg L^-1^ nitrogen, 80 mg L^-1^ phosphorus, and 60 mg L^-1^ potassium from Wuxal^®^ Super 8-8-6 plus micro liquid fertilizer (Aglukon). Petunia was transformed as described by Jorgensen et al. (1996), modified to remove acetosyringone from the co-cultivation medium.

### 2.2 Genotyping and ddPCR gene/allele copy counting

We used PCR-based analysis to determine the genotypes of particular plants as necessary. In most cases presence/absence of amplification was used to determine genotype, but direct sequencing of PCR products was also used. The primers used are given in the **Supplemental Table 1**. In some cases, we used ddPCR to quantify the number of transgenes/alleles that were present. Briefly, a reference gene in the nuclear genome was selected and used as a standard for two copies per genome (either *PhMAX1* or *PhCCD7*). Target genes, either *nptII* (as a measure of transgene copies) or *DAD1* (to test for homozygosity at the locus) were then measured against this. For these analyses we prepared genomic DNA using the Plant DNA II kit (Macherey-Nagel). The reactions were made up in 1x QX200™ ddPCR™ EvaGreen^®^ Supermix and the droplets created using an AutoDG™ droplet generator before PCR in a C1000 machine and detection in a QX200 droplet reader (Bio-Rad Laboratories). The data were analyzed using the QXManager software (v1).

### 2.3 Cloning

All primers described in this section are given in **Supplemental Table 1**. PCR (KAPA3G Plant Taq polymerase, KAPA biosystems) was used to isolate the *AtD14* promoter (*D14pro*) from genomic DNA purified from *Arabidopsis thaliana* (Plant DNA II, Macherey-Nagel). The resulting DNA fragment was cloned into the pXCB1 plasmid using restriction cloning (SacI, XhoI, NEB) to make the pXCB1-*D14pro* plasmid. The pXCB1 plasmid was derived from the pHEX2 plasmid (Hellens et al., 2005) by removing the CaMV 35S promoter. The DAD2recode coding sequence was designed in Geneious (Biomatters) using the codon optimization function and synthesized (Genscript). The mutants of DAD2 or DAD2recode were made by site directed mutagenesis using QuikChange Lightning (Agilent). The 10xMyc tag encoding DNA was PCR amplified from AB294444 (Nakagawa et al., 2007) and InFusion (Takara) cloned to DAD2 and DAD2recode. Gateway cloning (Invitrogen) was used to assemble the various DAD2 constructs in the pHEX2 or pXCB1-*D14pro* plasmids.

### 2.4 CRISPR/Cas9 method

The *AtD14* gene genomic sequence was tested for Cas9 target sites using the N(20):NGGH recognition motif in the Geneious (Biomatters) CRISPR design tool. The SpCas9 sgRNAs were created *in silico* and tested for secondary structure using the Multilign web service (Bellaousov et al., 2013). We selected four guide RNAs and created four single guide plant transformation plasmids based on pDE-SpCas9 by restriction cloning (Fauser et al., 2014). The *bar* selection cassette in pDE-SpCas9 was replaced with the *nptII* selection cassette from pDE-Cas9D10A by restriction cloning to create pDE-SpCas9-KanR. Each plasmid was independently transformed into *A. thaliana* Col-0 plants. Transgenic plants were selected on kanamycin and were screened for phenotypic changes and by DNA sequencing of PCR products spanning the target sites. Only guides 2 and 4 produced detectable editing in the T1 generation. A T1 plant line carrying the guide 2 construct was self-crossed twice to remove the T-DNA and bring the mutation to homozygosity, producing the *Atd14-7* plant line. The *Atd14-7* plant line is in the process of being submitted to ABRC.

### 2.5 Method for measuring plant leaf and overall plant area

Images of individual plants were recorded by digital photography. The location of the camera was directly above the plants, recording the total visible surface of the plant as viewed from this position. The plants within each image were identified by color space matching using the opencv library in Python version 3. Pixels identified as containing part of the plant were counted. Pixel counts were converted to cm^2^ by reference to a standard. The final values are referred to as the total leaf area or total plant area in the main text and figures.

### 2.6 Graphing and statistical analyses

Statistical analyses were performed using the Genstat statistical software package (22th Edn). ANOVAs were performed for statistical analyses of phenotypic and expression data. Appropriate transformations were used where necessary to ensure that model assumptions were met. Mean separation tests were performed using Tukey’s least significant difference (LSD) test or by Fisher’s protected LSD tests, both at the 5% level of significance. For any given character in an experiment, values with no common lowercase letter are significantly different from each other. Graphs are drawn with Graphpad Prizm version 9.

## 3 Results

### 3.1 Producing a functional strigolactone receptor in *Arabidopsis* based on the petunia orthologue required careful selection of the promoter and the coding sequence

**Figure 1:**
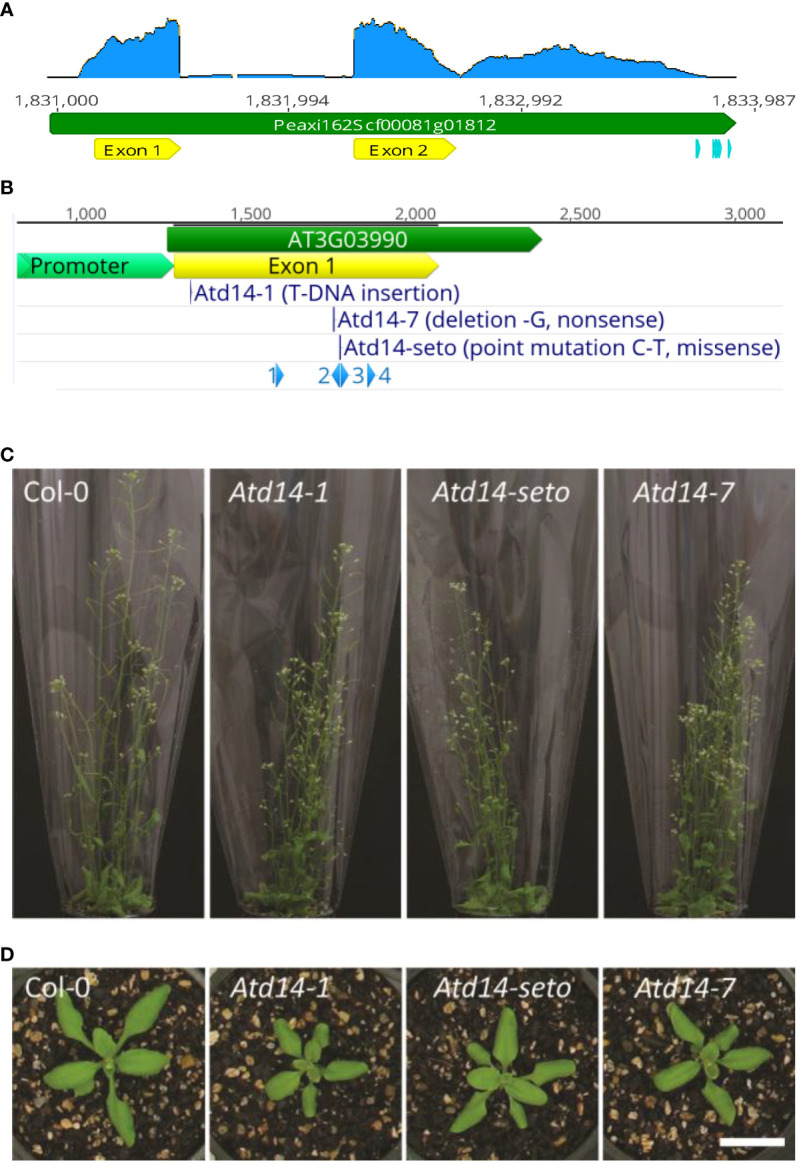
**(A)**, the structure of the *DAD2* genomic locus. The predicted gene model from the *Petunia axillaris* genome for the *DAD2* gene is Peaxi162Scf00081g01812; the gene (green) and CDS (yellow) are shown. Potential PolyA sites are shown (light blue). The trace in blue plots the read depth of the mRNAseq data that map to this region of the genome. **(B)**, the structure of the *AtD14* genomic locus. The predicted gene model from the publicly available *Arabidopsis thaliana* genome (arabidopsis.org) for the *AtD14* gene is At3g03990; the gene (green) and CDS (yellow) are shown. The location and nature of the mutants used in this work are shown in dark blue. The putative promoter region used in this work is shown (light green). The CRISPR-Cas9 guide sites used during the creation of the *Atd14-7* mutant are shown in light blue. **(C, D)**, representative images of the wild type (Col-0) and AtD14 mutants used in this study. **(C)** showing height and branching at 28 days after sowing. **(D)** showing rosettes at 15 days after sowing. Scale bar is 2 cm.

The protein coding sequence of *DAD2* is 804 bp in size and contains a single intron. Recently generated RNA-seq data from petunia axillary buds were used to examine the transcript produced from the *DAD2* gene (Luo et al., unpublished). Using the publicly available petunia genome for *Petunia axillaris* (Bombarely et al., 2016), we found evidence that the transcript comprises the predicted protein coding sequence with extensions to produce both a short 5’-UTR and an unexpectedly long 3’-UTR in the final mRNA. Very few reads were found to map to the predicted intron, suggesting that it is efficiently cleaved from the final mRNA form (**Figure 1A**). The pattern of RNAseq read depth is unusual, with the read depth dropping to near zero immediately following the CDS stop codon before increasing again, reaching a depth of half that of the CDS maximum 400–500 bp downstream (**Figure 1A**). The drop in read depth may simply be due to the two microsatellites (13 cytosines and 13 adenines) contained within this region of the DNA and the difficulty of sequencing and mapping fragments with these features. The role that the 3’ UTR plays in the function of the gene is unknown.

We used the well-established *Arabidopsis* system for transformation and phenotypic characterization to screen multiple gene mutants in multiple genetic backgrounds. Prior to this work a T-DNA mutant knockout and an EMS missense mutant for the AtD14 gene were widely available (Waters et al., 2012; Chevalier et al., 2014). To generate an *AtD14* knockout without an existing T-DNA and antibiotic resistance gene, we created a CRISPR/Cas9-mediated *AtD14* gene knockout. In the T1 generation a heterozygous CRISPR/Cas9-induced mutation was identified following transformation. In the T3 generation we identified a homozygous *AtD14* line containing a non-sense mutation at position 487 of the coding sequence (corresponding the CRISPR target 2) with no remaining detectable T-DNA, hereafter referred to as *Atd14-7* (**Figure 1B**). Comparisons between the wild type and the existing mutants and the new *Atd14-7* mutant suggested all the mutants had similar reduced height, increased branching and smaller rosettes (**Figures 1C, D**; **Supplemental Figures 2B–F**). We confirmed the wild type state of the five most likely off-target editing sites (**Supplemental Figure 1A**). To produce a double mutant that was expected to be both strigolactone-deficient and strigolactone-insensitive whilst still sensitive to kanamycin selection, we crossed the newly created *Atd14-7* mutant line with the *max4-2* mutant line (Tissier et al., 1999; Sorefan et al., 2003) and confirmed mutant allele identity by PCR-based genotyping (**Supplemental Figure 1B**).

**Figure 2:**
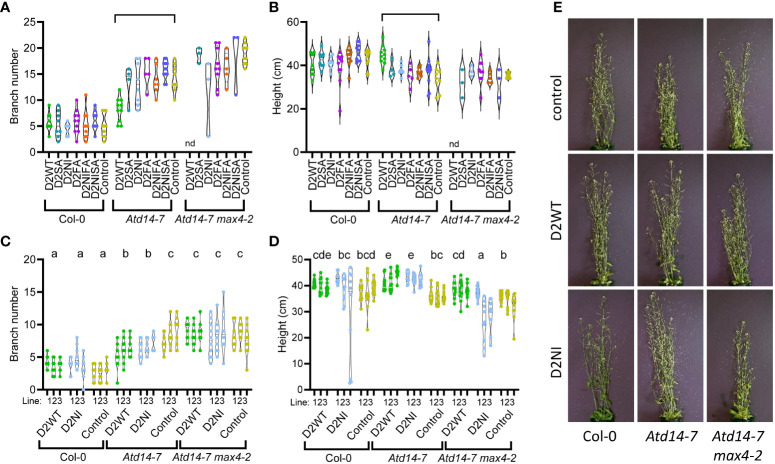
Phenotypic analysis of *A. thaliana* plants (Col-0, *Atd14-7* and *Atd14-7 max4-2* genotypes) transformed with various recoded DAD2 (wild type and mutant) genes expressed from the *AtD14* promoter. Graphs are violin plots showing median and quartiles. Points are individual data. **(A, B)** The rosette branch number and the height of primary transformant plants were measured at 61 days after sowing. The plants were selected on kanamycin before being transferred to a growth room. D2WT = wild type CDS, D2SA = S96A mutation, D2NI = N241I mutation, D2FA = F135A mutation, D2NIFA = N242I and F135A mutations, D2NISA = N242I and S96A mutations, Control = empty plasmid. (n=3-12). nd, not determined. Within each graph the braces indicate pairs of data that are significantly different by Tukey’s LSD tests (α = 0.05). A full list of the means and the results from the statistical test are given in **Supplemental Table 2**. **(C, D)** The rosette branch number and the height of three T2 lines from independent transformation events for the Control, D2WT and D2NI genes were measured at 44 days after sowing. The plants were selected on kanamycin before being transferred to the growth room (n=10). Within each graph groups of data that share a lowercase letter are not significantly different by Tukey’s LSD tests (α = 0.05). **(E)** Representative plants from the T2 dataset, images taken at the time of measurement.

In previous work we showed that the *DAD2* gene’s protein coding sequence alone (UTRs and intron removed) was sufficient to complement many of the petunia *dad2* mutant phenotypes when expressed from a CaMV 35S constitutive promoter (Hamiaux et al., 2012). As part of testing the function of the *DAD2* gene in *Arabidopsis*, we tried a number of synthetic gene constructs that used variations on the *DAD2* gene and its transcriptional cassette (**Supplemental Figure 2A**). Using a CaMV 35S promoter, an epitope tag and even the native petunia coding sequence on the *Arabidopsis* endogenous *AtD14* promoter, we were not able to reproduce functional strigolactone receptor activity (**Supplemental Figures 2B–F**). Finally, we generated a petunia *DAD2* coding sequence that had been recoded by using a codon optimization process that led to altering 125 of 288 codons (**Supplemental Figure 2A**, **Supplemental Data 1**) with no epitope tags and expressed this gene from 731 bp of the native *AtD14* gene’s promoter region. Only when using this gene construct were we able to detect a change in the phenotype of *Atd14-7* plants suggestive of any functional complementation of the lost *AtD14* gene (**Figures 2A, B**).

Having shown that the wild type petunia DAD2 protein was able to reduce branching of the *Atd14-7* mutant, we wanted to investigate the function of DAD2 and its mutants, specifically DAD2-N242I, DAD2-F135A, DAD2-S96A and the double mutants DAD2-N242IF135A and DAD2-N242IS96A. We hypothesized that these mutants might more strongly inhibit branching in Col-0 and/or be able to provoke some signaling even if no strigolactone was produced in the *max4-2* plants. In a screen of T1 transgenic plants transformed with the recoded version of *DAD2*, the wild type protein could partially complement the *Atd14-7* mutant. As expected this did not affect the phenotype of the wild type Col-0 control (**Figures 2A, B**). However, none of the mutant *DAD2* constructs produced statistically significant changes to the branching or height of the plants in this generation for either the Col-0 wild type or the *Atd14-7* or the *Atd14-7max4-2* genetic backgrounds. Nonetheless, a subset of the *Atd14-7* plants transformed with the DAD2-N242I construct appeared to have decreased branching. To characterize the effect of the wild type DAD2 and mutant DAD2-N242I constructs on the growth of *Arabidopsis*, three independent transformed lines carrying a single transgenic locus for each genetic background were taken to the T2 generation and their growth characteristics analyzed after preselection of plants on kanamycin (**Figures 2C–E**). In this generation, each of the three independent lines carrying the wild type DAD2 in each of the three backgrounds produced the predicted result: the branching and height of the lines was altered only for the *Atd14-7* mutant lines. This confirms that the wild type DAD2 protein can complement for the loss of the endogenous AtD14 protein, but suggests it still requires strigolactone (produced by the MAX4 protein) to function in the signaling pathway. Plant lines transformed with the DAD2-N242I transgene also had reduced branching and increased height in the *Atd14-7* background (**Figures 2C–E**). However, as for DAD2-WT, no changes to branch number were seen in the Col-0 or the *Atd14-7max4-2* backgrounds.

The difference between petunia DAD2 before and after recoding the nucleotide sequence in their ability to complement *Arabidopsis AtD14* mutants was unexpected and led us to analyze the RNA transcripts that were produced from the introduced native version of *DAD2* gene in *Arabidopsis*. We detected two mis-spliced variants of the petunia gene. The first transcript is spliced from base 592 to base 933 and the other from base 611 to 933, as indicated by the mixed sequence trace from base 592 to base 611 (**Supplemental Figure 3**). The forms produced are suggestive of conserved intron defining sequences in the petunia *DAD2* coding sequence that are inappropriately but reliably detected by the *Arabidopsis* splicing machinery.

### 3.2 The phenotype of petunia *dad2* plants is altered by a constitutively expressed *DAD2* transgene such that these plants are neither wild type nor *dad2* like

Following the initial difficulty of the complementation work in *Arabidopsis*, we began parallel transformations of petunia with similar constructs. We produced 5–7 independent transformants for each of the four transgenes DAD2-WT, DAD2-N242I, DAD2-F153A and DAD2-N242IF153A in the *dad2* mutant background. For these experiments we expressed the *DAD2* coding sequence (both WT and mutants) using the CaMV 35S promoter, given similar constructs had been shown to complement the mutant *dad2* phenotype in previous work with primary transformants (Hamiaux et al., 2012). Homozygous T3 plants were produced for at least three independent transgenic lines for each construct. Each of these lines was crossed to a *dad1dad2* double mutant line. Each transgene was again brought to homozygosity in a homozygous *dad2* and *dad1dad2* background in the T4 generation. We qualitatively assessed the phenotypes of the plant lines in each generation and quantitively assessed the phenotypes in the T2 and T5 generations.

In the T1 generation, qualitative assessment of transgenic plants indicated that phenotypes had been altered by the addition of DAD2 wild type and mutant transgenes tested here. However, further analysis was confounded by the impacts of growth in tissue culture, presence of selection and the varying time between the initiation of transgenesis and the final production of plants in the glasshouse.

**Figure 3:**
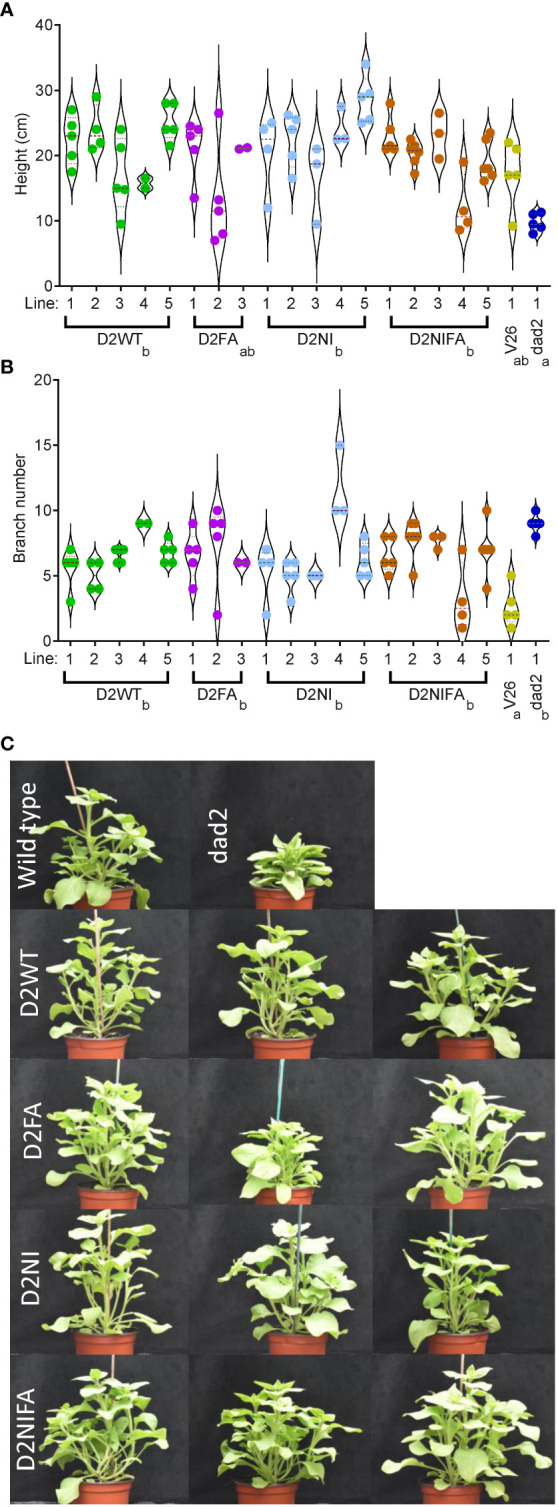
Phenotypic analysis of mutant petunia plants (*dad2*) transformed with various DAD2 (wild type and mutant) genes expressed from the CaMV *35S* promoter. D2WT = wild type CDS, D2FA = F135A mutation, D2NI = N241I mutation, D2NIFA = N242I and F135A mutations. An untransformed wild type (V26) and mutant line (*dad2*) are shown for comparison. Graphs are violin plots with median and quartiles indicated, points are individual data. n = 2–6. Within each graph genotypes that share a lowercase identifier are not significantly different by Tukey’s LSD tests (α = 0.05). **(A)**, height of the plants. **(B)**, number of branches longer than 2 cm. **(C)**, images of plants from the experiment in **(A, B)**. The top row are the control wild type and *dad2* lines. The remaining rows show plants from three different transgenic lines for each construct in the *dad2* background.

In the T2 generation, we grew 3–5 transgenic lines and controls in a single glasshouse room over an eight-week period in spring. We grew six plants from each line, genotyped these for the transgene and quantitatively assessed the phenotypes of the transgene-positive plants. The DAD2-WT, DAD2-NI and DAD2-NIFA transgenes increased the heights of the plants compared with the *dad2* control plants (**Figure 3A**). Surprisingly, we did not see a statistically significant decrease in branching for any transgene in this generation despite some individual plants appearing dramatically different from *dad2* control plants (**Figures 3B, C**).

In the T4 generation, we not only qualitatively assessed the effect of the transgenes on the *dad2* mutant plants but could also examine the effects of these genes on the double mutant *dad1dad2* plants. As expected, the wild type *DAD2* gene and the mutant genes were able to alter the branching and height characteristics of only the *dad2* mutant plants (**Supplemental Figure 4**). As was observed in the *Arabidopsis* work, none of the transgenes were able to alter the *dad1dad2* phenotypes. This strongly suggests that the mutant proteins do not constitutively interact with their binding partners (leading to signal transduction without SL) despite the yeast-2-hybrid data suggesting this could be possible (Lee et al., 2020).

**Figure 4:**
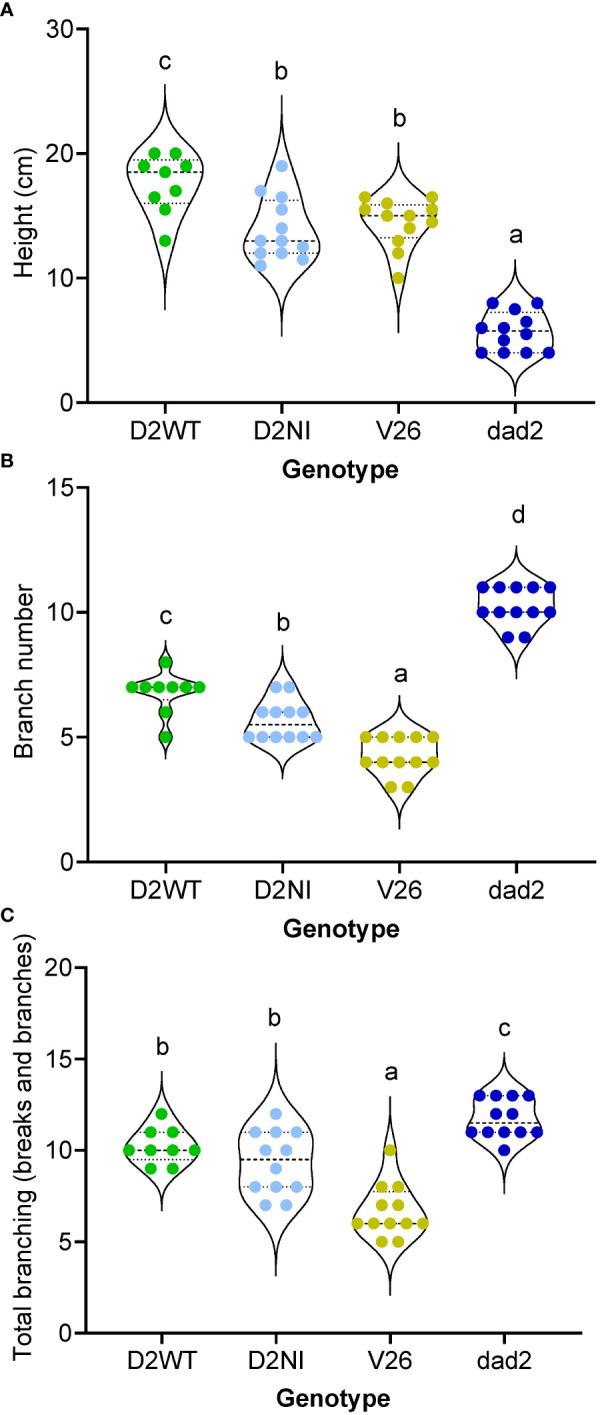
Phenotypic analysis of mutant petunia plants (*dad2*) transformed with D2WT = DAD2 wild type CDS, and D2NI = DAD2 with the N241I mutation. An untransformed wild type (V26) and mutant line (*dad2*) are shown for comparison (n= 9–12). The measurements were made at 51 days post sowing. Graphs are violin plots with median and quartiles indicated, points are individual data. Within each graph data with the same lowercase identifier are not significantly different by Fisher’s protected least significant difference tests **(A)**, height of the plants. **(B)**, number of branches longer than 2 cm. **(C)**, number of axillary buds with at least two leaves.

A T5 generation of seeds were generated by self-crossing T4 plants that contained single copy homozygous transgenic events and that were also homozygous for the *dad2-3* allele (as determined by ddPCR). Using these plant lines, we sought to further refine our understanding of the wild type DAD2-WT and DAD2-N242I transgenes’ function. Despite the careful selection of lines homozygous for single insertion transgenic events, our DAD2-WT line produced three plants (25%) with characteristic *dad2* phenotypes. As these plants still carried the transgene, as determined by PCR, perhaps silencing of the construct had occurred in these plants. The data from these plants are recorded in the **Supplemental Data Files** but are excluded from the analyzed data presented in the figures. The wild type *DAD2* transgene was capable of increasing the height of *dad2* plants to an extent that these plants were taller than the wild type V26 controls (**Figure 4A**). At the same time, the number of branches was intermediate between the *dad2* and V26 values (**Figure 4B**). When “breaks” (Snowden and Napoli, 2003) were included, the number of axillary buds showing some growth in the *dad2* DAD2-WT line was more similar to that with *dad2*, suggesting that the transgene is not able to control bud growth initiation as tightly as the wild type gene (**Figure 4C**).

**Figure 5:**
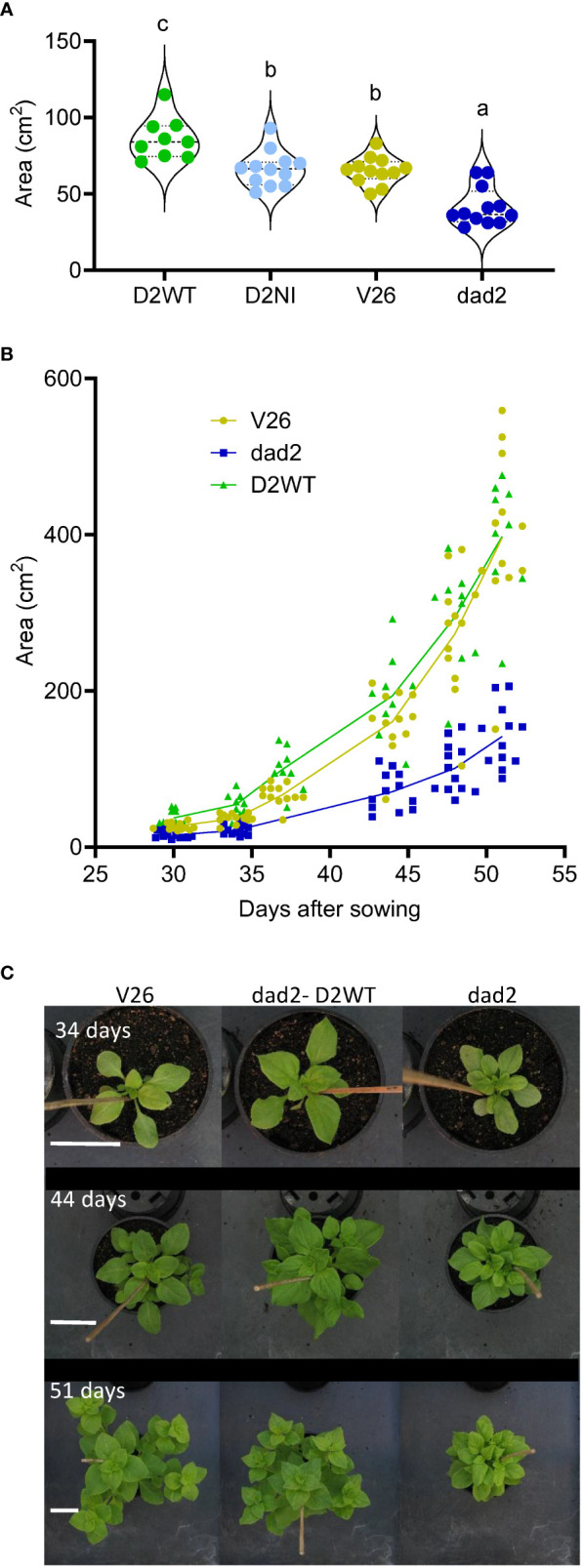
Phenotypic analysis of mutant petunia plants (*dad2*) transformed with D2WT = DAD2 wild type CDS, and D2NI = DAD2 with the N241I mutation. An untransformed wild type (V26) and mutant line (*dad2*) are shown for comparison (n= 9–12). **(A)**, Combined area of leaves 5–8 on the main stem. Graph is a violin plot with median and quartiles indicated, points are individual data. Data with the same lowercase identifier are not significantly different by Fisher’s protected least significant difference tests **(B)**, Total plant area graphed as a function of days after sowing, all data shown, lines show the change in mean over time **(C)**, Images of representative plants from 34, 44 and 51 days. Scale bars (white) are 5 cm and apply to images in the same row.

Other vegetative traits were also examined in the T5 plants. In earlier work we identified that the leaves of both the *dad1* and *dad2* mutants are smaller overall, with decreased length, but increased blade to petiole length ratios compared with those of wild type V26 (**Supplemental Figure 5**). Previous work has shown that in *Arabidopsis*, *Medicago truncatula* and *Festuca arundinacea*, leaf morphology traits are altered by changes in SL pathway signaling, although the change is quite different in each species (Stirnberg et al., 2002; Lauressergues et al., 2015; Hu et al., 2019). To further understand the changes in petunia leaf morphology we made measurements of the fifth to eighth leaves. The V26 and *dad2* mutant leaves were significantly different for each of leaf size, blade length, petiole length and blade width and the ratio of blade length to petiole length (**Figure 5A**; **Supplemental Figure 6**). The DAD2-WT transgene increased the *dad2* leaf dimensions. However, the increase was uneven and resulted in a leaf shape and size different from those of both V26 and *dad2* leaves (**Figure 5A**; **Supplemental Figure 6**). We further used image analysis and quantification to show that the total plant area of the *dad2* DAD2-WT plants was increased compared with that of V26 (**Figures 5B, C**). Computer-aided image analysis of the plants showed that the growth of the *dad2* DAD2-WT plants was greater in the earlier part of the experiment, but that the wild type V26 plant quickly produced plants of the same measured size (**Figure 5B**), probably because of self-masking of leaves in the more compact *dad2* DAD2-WT plants. We observed that the *dad2* DAD2-WT plants retained a more compact and radially symmetric form – see top-down images of the plants over time (**Figure 5C**) showing these plants retained a form more like that of the *dad2* plants, contrasting with the V26 wild type plants, which had a star-shaped form created by the lateral extension of the largest branches (**Figure 5C**).

## 4 Conclusions and discussion

We have extended our analysis of selected DAD2 mutations that were characterized biochemically in Lee et al. (2020). Prior work showed that certain DAD2 mutant proteins were able to interact with signaling partners, in yeast 2-hybrid experiments, even in the absence of SL. Whilst the wild type gene and the tested mutants were functional in both plant species by at least partially complementing the respective strigolactone receptor mutants, we were unable to detect significant strigolactone-independent functionality of these mutants *in planta*. We were able to demonstrate that the regulation of the expression of the strigolactone receptor in both petunia and *Arabidopsis* influences the control of the core strigolactone characteristics of plant height and branching. We also showed that in petunia there were leaf shape as well as plant size and shape changes.

Expressing the mutant *DAD2* genes identified in Lee et al. (2020) did not complement the SL negative/SL-insensitive double mutant plants. Potentially the differences could simply have come from the use of different experimental systems (yeast versus plants). However, in the *Arabidopsis* system, the DAD2 binding partners MAX2 and AtD53 appear to be capable of interacting with the petunia DAD2 wild type protein, as complementation of the *Atd14-7* mutant phenotype was possible. However, there are some sequence differences compared with the petunia orthologues which may explain the lack of phenotypic effects when we used the mutated *DAD2* genes in *Arabidopsis*. In petunia however, the binding partners were exactly those in the yeast experiment. Whether the proteins do not interact, or whether upon interacting they are not in a form able to transduce the signal, requires further investigation. The phenotypic output could additionally be mediated by unknown proteins present in plants that act redundantly of the DAD2/PhMAX2/PhD53A complex to control the activity of the SL signaling system.

Expressing the wild type *DAD2* coding sequence under the control of the CaMV 35S promoter did not entirely complement the *dad2* mutant phenotype. The expression pattern of the strigolactone receptor is one mechanism that influences its effects (Arite et al., 2009; Umehara et al., 2010; Hamiaux et al., 2012; Waters et al., 2012; Guo et al., 2013; Chevalier et al., 2014; Drummond et al., 2015). Our previous work indicated that the transcript abundance of the *DAD2* gene was altered in response to environmental inputs in petunia (Drummond et al., 2015). In this study we removed most, if not all, of the native regulatory structure from the *DAD2* gene. The expression patterns of the CaMV 35S promoter are relatively uniform at macroscopic scales throughout the plant and are largely unaffected by environmental inputs (Odell et al., 1985; Holtorf et al., 1995). However, it is considered that the activity of the CaMV 35S promoter is reduced in reproductive cells and in apical meristems (Sunilkumar et al., 2002). Here the use of the 35S promoter driving expression of *DAD2* generated plants with changes to overall branch number, plant shape/compactness, leaf size, and height that were distinct from both wild type and the *dad2* mutant phenotypes. Similarly to the CaMV 35S promoter in dicots the *ACTIN1* promoter can be used in rice to express genes in a wide range of cells and tissues (Zhang et al., 1991). The expression of the rice *DAD2* orthologue *D14* from the *ACTIN1* promoter in a wild type genetic background increased branching beyond wild type (Patil et al., 2022). The increased branching in the rice system suggests the possibility that in our petunia experiments the expression of *DAD2* has complemented the mutant allele but additionally triggered additional branching by some other mechanism. Further experiments would be required to separate these two possibilities. Although the most obvious characteristic SL modulates is that of plant branching, it also appears that SL is used to fine-tune the expression of other phenotypic characters and this is, at least in part, through regulation of the gene’s transcription.

Petunia and *Arabidopsis* SL mutants have smaller and rounder leaves than their respective wild type plants. The roundness comes from the disproportionate decrease in length compared with the width of the leaves in these plants (Stirnberg et al., 2002; **Supplemental Figure 6**). In petunia the decrease in petiole length is more severe than the decrease in the blade length. More interestingly, the DAD2-WT transgene increased the leaf size beyond that of the wild type by increasing both blade width and length, although only partially restoring the petiole length. One possible explanation is that the expression of *DAD2* is increased beyond that of wild type, potentially leading to increased sensitivity to SL. This is consistent with the results in *F. arundinacea*, where leaf length was increased by the addition of the SL analogue GR24 (Hu et al., 2019). However, in *M. truncatula*, SL mutation affects the complexity of the margins of the leaves, (Lauressergues et al., 2015), which was not observed here in petunia.

Regulating the expression of the SL receptor is likely to provide an avenue for generation of novel phenotypes. The observed changes in the phenotype of petunia plants suggest manipulation of the strigolactone receptor and in particular the localization of receptor expression will enable us to design plants with architectures that may suit a range of different agricultural settings, including controlled environment agriculture. One potentially interesting avenue of research would be to further deconstruct the expression of the strigolactone receptor by one of two routes: either via CRISPR/Cas-mediated deconstruction and permutation of the petunia DAD2 promoter region *in situ* (Rodríguez-Leal et al., 2017), or via synthetic biology refactoring of the entire gene using transcript recoding and genomic region complementation approaches (Brophy, 2022). This second approach might allow gene function interrogation of the intron and UTRs. Finally, the use of either tissue-specific or combinatorial synthetic domain localization methods would provide another route to examining the effect(s) when strigolactone response is localized to specific organs (Brophy et al., 2022).

## Data availability statement

The original contributions presented in the study are included in the article/**Supplementary Material**. Further inquiries can be directed to the corresponding authors.

## Author contributions

RD: Conceptualization, Data curation, Formal Analysis, Investigation, Methodology, Supervision, Visualization, Writing – original draft, Writing – review & editing. HL: Formal Analysis, Investigation, Visualization, Writing – review & editing. ZL: Investigation, Writing – review & editing. JD: Investigation, Writing – review & editing. BJ: Funding acquisition, Investigation, Supervision, Writing – review & editing. KS: Conceptualization, Data curation, Formal Analysis, Funding acquisition, Investigation, Project administration, Supervision, Writing – review & editing.
