# Supplementary material for: Varying the expression pattern of the strigolactone receptor gene DAD2 results in phenotypes distinct from both wild type and knockout mutants
Source: Front Plant Sci. 2023 Oct 11;14:1277617. doi: 10.3389/fpls.2023.1277617 (PMC10600376; doi:10.3389/fpls.2023.1277617)
Supplement: Supplementary file 2 [file Presentation_1.pptx]

## Slide 1
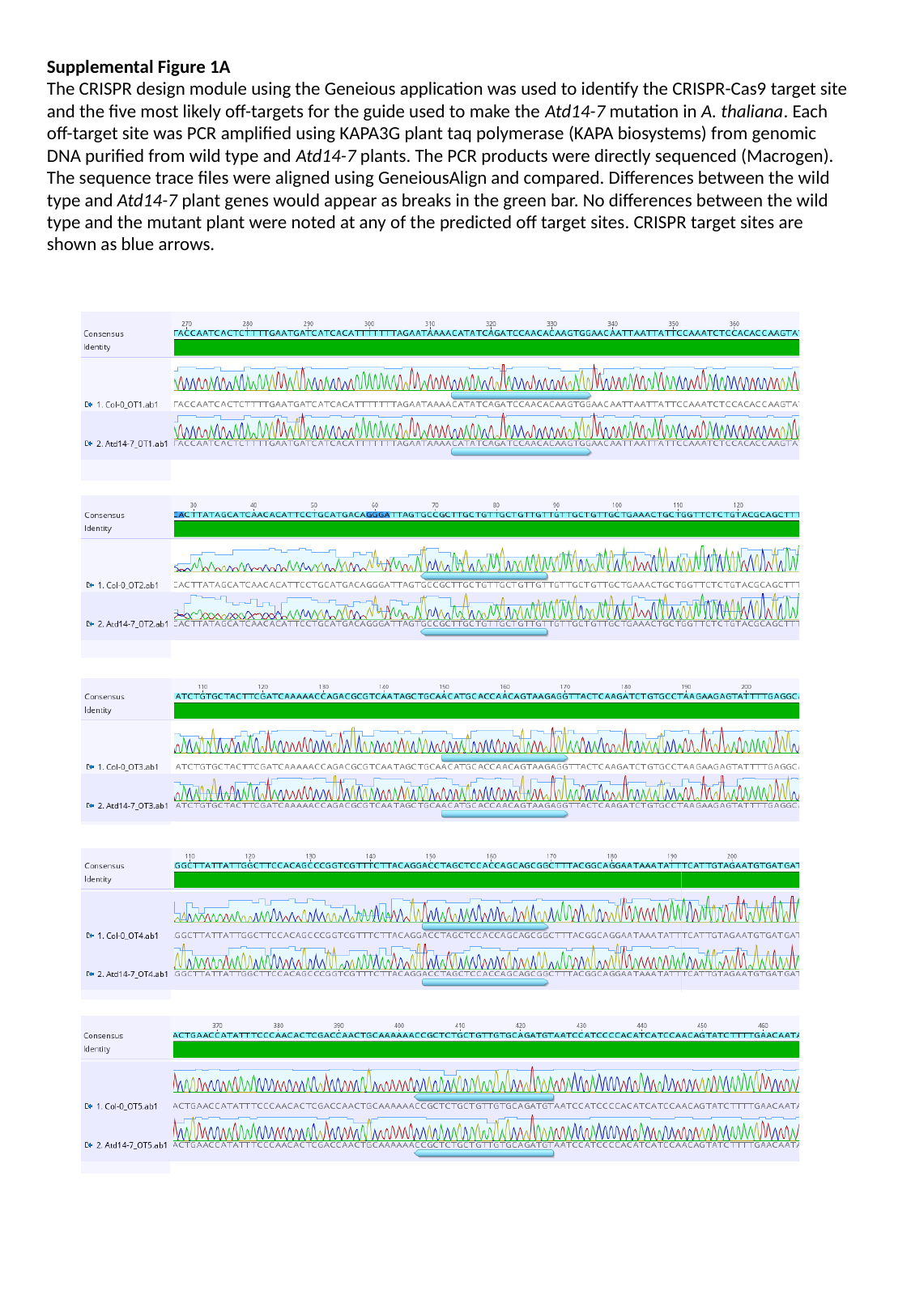

Supplemental Figure 1A
The CRISPR design module using the Geneious application was used to identify the CRISPR-Cas9 target site and the five most likely off-targets for the guide used to make the Atd14-7 mutation in A. thaliana. Each off-target site was PCR amplified using KAPA3G plant taq polymerase (KAPA biosystems) from genomic DNA purified from wild type and Atd14-7 plants. The PCR products were directly sequenced (Macrogen). The sequence trace files were aligned using GeneiousAlign and compared. Differences between the wild type and Atd14-7 plant genes would appear as breaks in the green bar. No differences between the wild type and the mutant plant were noted at any of the predicted off target sites. CRISPR target sites are shown as blue arrows.

## Slide 2
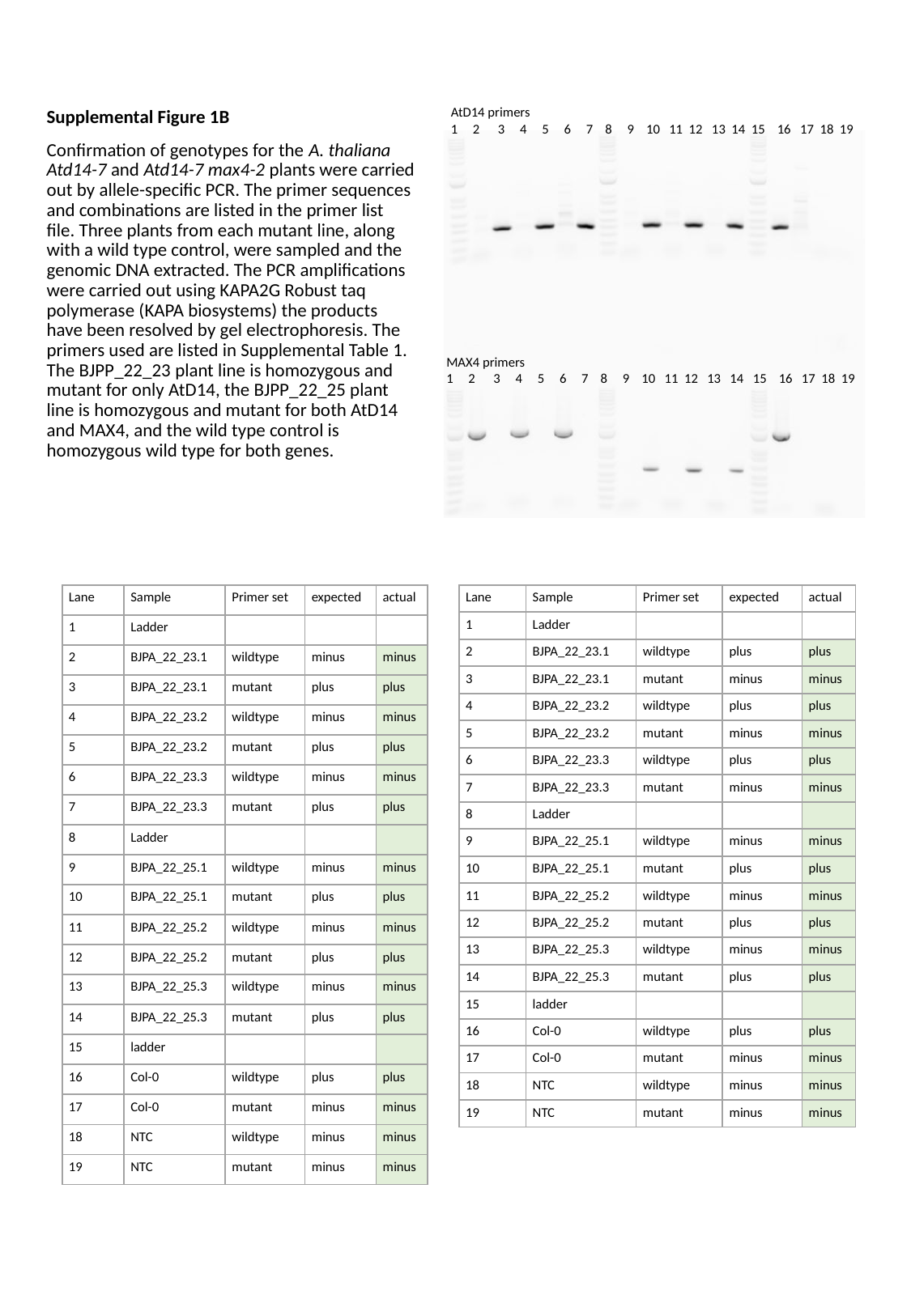

AtD14 primers
1 2 3 4 5 6 7 8 9 10 11 12 13 14 15 16 17 18 19
Supplemental Figure 1B
Confirmation of genotypes for the A. thaliana Atd14-7 and Atd14-7 max4-2 plants were carried out by allele-specific PCR. The primer sequences and combinations are listed in the primer list file. Three plants from each mutant line, along with a wild type control, were sampled and the genomic DNA extracted. The PCR amplifications were carried out using KAPA2G Robust taq polymerase (KAPA biosystems) the products have been resolved by gel electrophoresis. The primers used are listed in Supplemental Table 1. The BJPP_22_23 plant line is homozygous and mutant for only AtD14, the BJPP_22_25 plant line is homozygous and mutant for both AtD14 and MAX4, and the wild type control is homozygous wild type for both genes.
MAX4 primers
1 2 3 4 5 6 7 8 9 10 11 12 13 14 15 16 17 18 19
| Lane | Sample | Primer set | expected | actual |
| --- | --- | --- | --- | --- |
| 1 | Ladder | | | |
| 2 | BJPA\_22\_23.1 | wildtype | minus | minus |
| 3 | BJPA\_22\_23.1 | mutant | plus | plus |
| 4 | BJPA\_22\_23.2 | wildtype | minus | minus |
| 5 | BJPA\_22\_23.2 | mutant | plus | plus |
| 6 | BJPA\_22\_23.3 | wildtype | minus | minus |
| 7 | BJPA\_22\_23.3 | mutant | plus | plus |
| 8 | Ladder | | | |
| 9 | BJPA\_22\_25.1 | wildtype | minus | minus |
| 10 | BJPA\_22\_25.1 | mutant | plus | plus |
| 11 | BJPA\_22\_25.2 | wildtype | minus | minus |
| 12 | BJPA\_22\_25.2 | mutant | plus | plus |
| 13 | BJPA\_22\_25.3 | wildtype | minus | minus |
| 14 | BJPA\_22\_25.3 | mutant | plus | plus |
| 15 | ladder | | | |
| 16 | Col-0 | wildtype | plus | plus |
| 17 | Col-0 | mutant | minus | minus |
| 18 | NTC | wildtype | minus | minus |
| 19 | NTC | mutant | minus | minus |
| Lane | Sample | Primer set | expected | actual |
| --- | --- | --- | --- | --- |
| 1 | Ladder | | | |
| 2 | BJPA\_22\_23.1 | wildtype | plus | plus |
| 3 | BJPA\_22\_23.1 | mutant | minus | minus |
| 4 | BJPA\_22\_23.2 | wildtype | plus | plus |
| 5 | BJPA\_22\_23.2 | mutant | minus | minus |
| 6 | BJPA\_22\_23.3 | wildtype | plus | plus |
| 7 | BJPA\_22\_23.3 | mutant | minus | minus |
| 8 | Ladder | | | |
| 9 | BJPA\_22\_25.1 | wildtype | minus | minus |
| 10 | BJPA\_22\_25.1 | mutant | plus | plus |
| 11 | BJPA\_22\_25.2 | wildtype | minus | minus |
| 12 | BJPA\_22\_25.2 | mutant | plus | plus |
| 13 | BJPA\_22\_25.3 | wildtype | minus | minus |
| 14 | BJPA\_22\_25.3 | mutant | plus | plus |
| 15 | ladder | | | |
| 16 | Col-0 | wildtype | plus | plus |
| 17 | Col-0 | mutant | minus | minus |
| 18 | NTC | wildtype | minus | minus |
| 19 | NTC | mutant | minus | minus |

## Slide 3
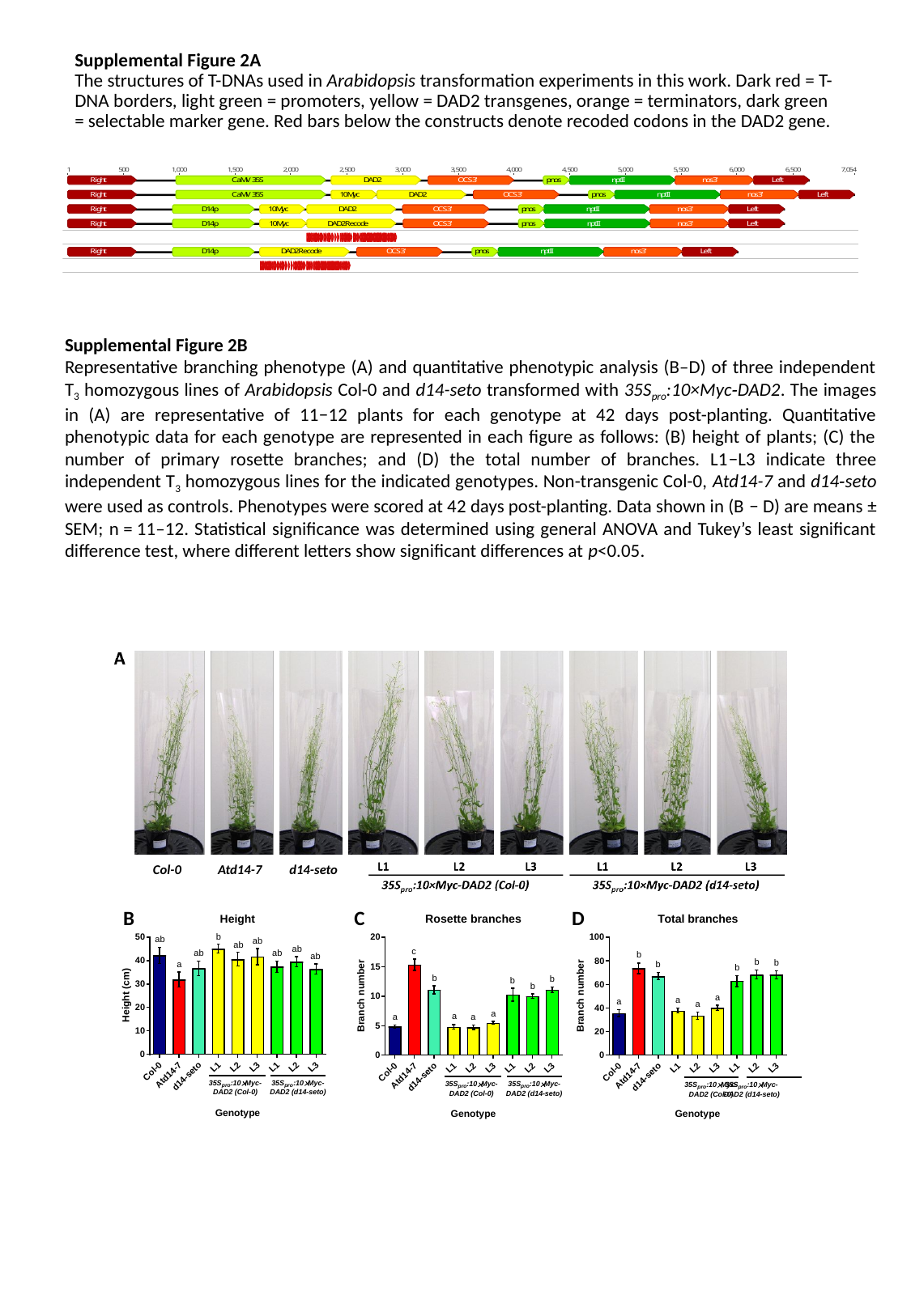

Supplemental Figure 2A
The structures of T-DNAs used in Arabidopsis transformation experiments in this work. Dark red = T-DNA borders, light green = promoters, yellow = DAD2 transgenes, orange = terminators, dark green = selectable marker gene. Red bars below the constructs denote recoded codons in the DAD2 gene.
Supplemental Figure 2B
Representative branching phenotype (A) and quantitative phenotypic analysis (B–D) of three independent T3 homozygous lines of Arabidopsis Col-0 and d14-seto transformed with 35Spro:10×Myc‑DAD2. The images in (A) are representative of 11−12 plants for each genotype at 42 days post-planting. Quantitative phenotypic data for each genotype are represented in each figure as follows: (B) height of plants; (C) the number of primary rosette branches; and (D) the total number of branches. L1−L3 indicate three independent T3 homozygous lines for the indicated genotypes. Non-transgenic Col-0, Atd14-7 and d14‑seto were used as controls. Phenotypes were scored at 42 days post-planting. Data shown in (B − D) are means ± SEM; n = 11–12. Statistical significance was determined using general ANOVA and Tukey’s least significant difference test, where different letters show significant differences at p<0.05.
Col-0 Atd14-7 d14-seto
B
C
D

## Slide 4
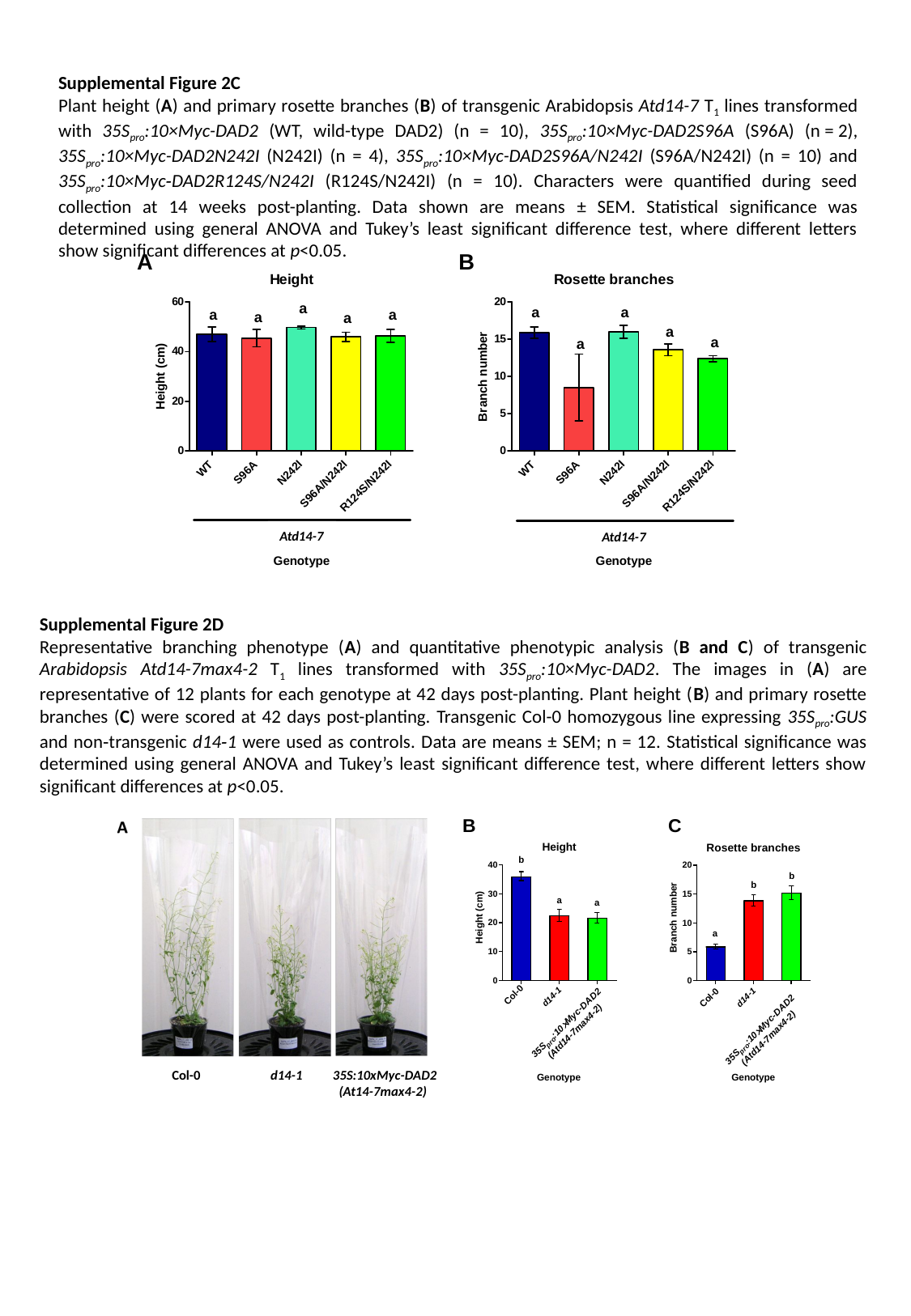

Supplemental Figure 2C
Plant height (A) and primary rosette branches (B) of transgenic Arabidopsis Atd14-7 T1 lines transformed with 35Spro:10×Myc-DAD2 (WT, wild-type DAD2) (n = 10), 35Spro:10×Myc-DAD2S96A (S96A) (n = 2), 35Spro:10×Myc-DAD2N242I (N242I) (n = 4), 35Spro:10×Myc-DAD2S96A/N242I (S96A/N242I) (n = 10) and 35Spro:10×Myc‑DAD2R124S/N242I (R124S/N242I) (n = 10). Characters were quantified during seed collection at 14 weeks post-planting. Data shown are means ± SEM. Statistical significance was determined using general ANOVA and Tukey’s least significant difference test, where different letters show significant differences at p<0.05.
Atd14-7
Atd14-7
Supplemental Figure 2D
Representative branching phenotype (A) and quantitative phenotypic analysis (B and C) of transgenic Arabidopsis Atd14-7max4-2 T1 lines transformed with 35Spro:10×Myc-DAD2. The images in (A) are representative of 12 plants for each genotype at 42 days post-planting. Plant height (B) and primary rosette branches (C) were scored at 42 days post-planting. Transgenic Col-0 homozygous line expressing 35Spro:GUS and non‑transgenic d14‑1 were used as controls. Data are means ± SEM; n = 12. Statistical significance was determined using general ANOVA and Tukey’s least significant difference test, where different letters show significant differences at p<0.05.
Col-0 d14-1 35S:10xMyc-DAD2
 (At14-7max4-2)

## Slide 5
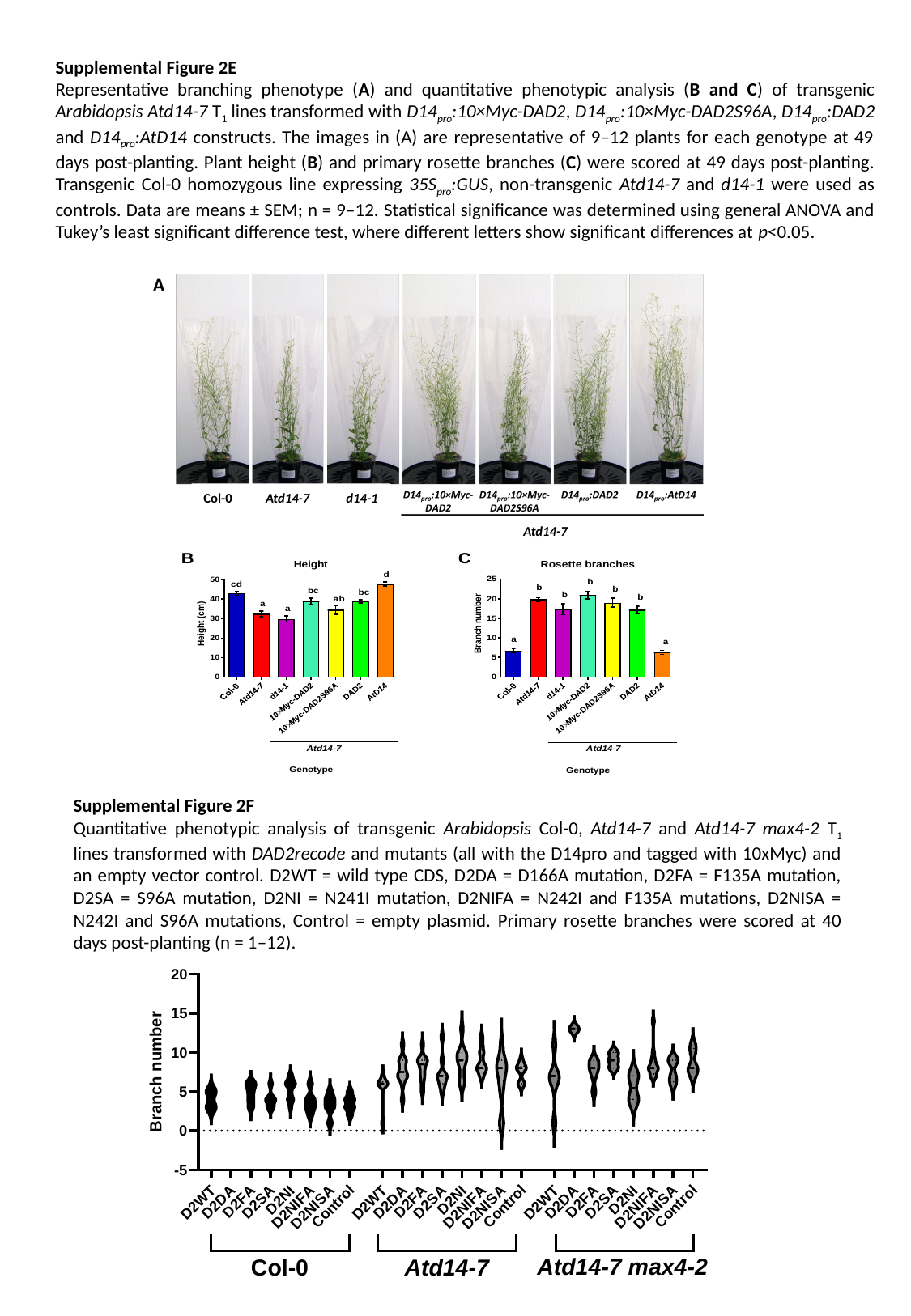

Supplemental Figure 2E
Representative branching phenotype (A) and quantitative phenotypic analysis (B and C) of transgenic Arabidopsis Atd14-7 T1 lines transformed with D14pro:10×Myc-DAD2, D14pro:10×Myc-DAD2S96A, D14pro:DAD2 and D14pro:AtD14 constructs. The images in (A) are representative of 9–12 plants for each genotype at 49 days post-planting. Plant height (B) and primary rosette branches (C) were scored at 49 days post-planting. Transgenic Col-0 homozygous line expressing 35Spro:GUS, non-transgenic Atd14-7 and d14-1 were used as controls. Data are means ± SEM; n = 9–12. Statistical significance was determined using general ANOVA and Tukey’s least significant difference test, where different letters show significant differences at p<0.05.
Col-0 Atd14-7 d14-1
Atd14-7
Supplemental Figure 2F
Quantitative phenotypic analysis of transgenic Arabidopsis Col-0, Atd14-7 and Atd14-7 max4-2 T1 lines transformed with DAD2recode and mutants (all with the D14pro and tagged with 10xMyc) and an empty vector control. D2WT = wild type CDS, D2DA = D166A mutation, D2FA = F135A mutation, D2SA = S96A mutation, D2NI = N241I mutation, D2NIFA = N242I and F135A mutations, D2NISA = N242I and S96A mutations, Control = empty plasmid. Primary rosette branches were scored at 40 days post-planting (n = 1–12).

## Slide 6
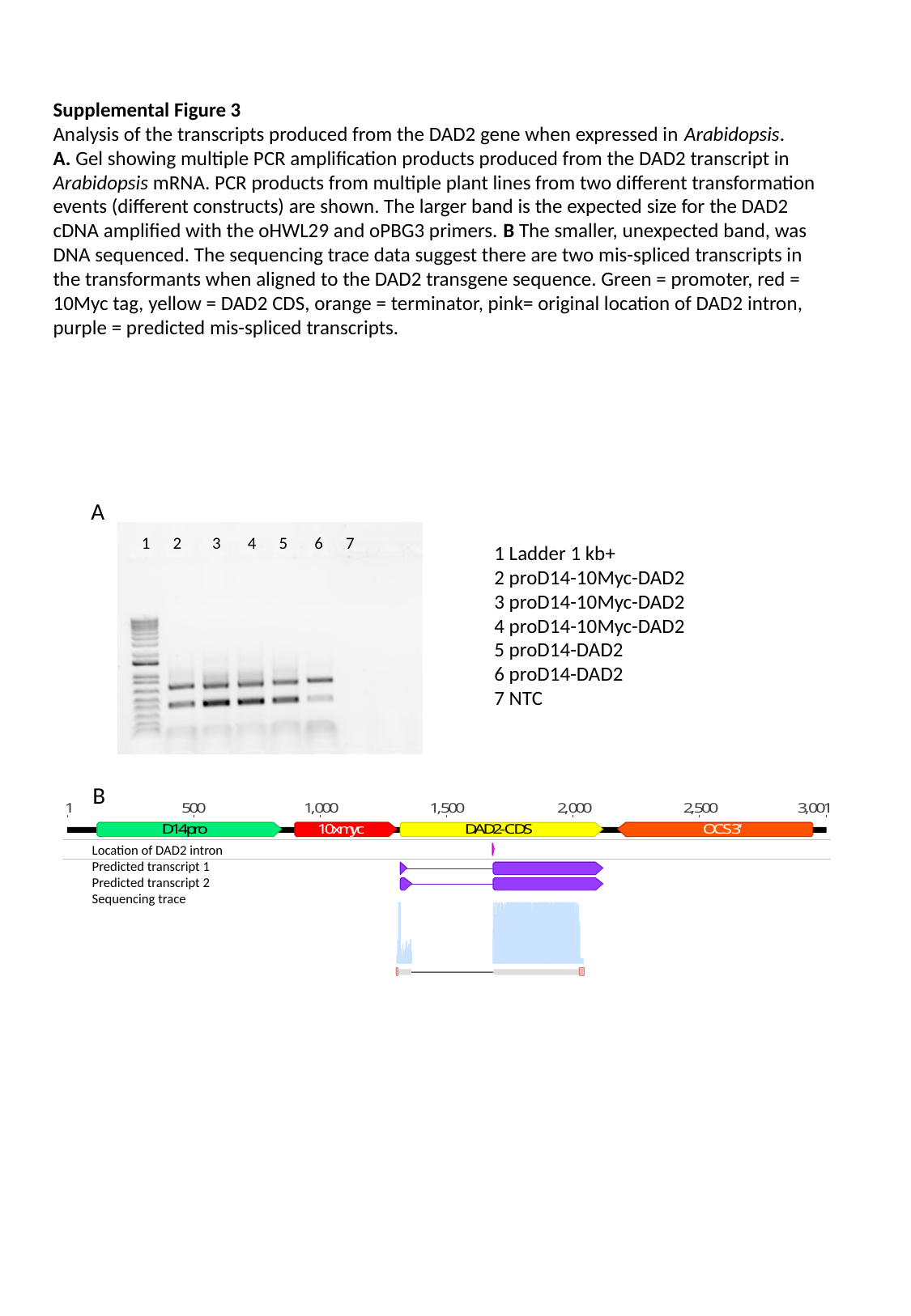

Supplemental Figure 3
Analysis of the transcripts produced from the DAD2 gene when expressed in Arabidopsis.
A. Gel showing multiple PCR amplification products produced from the DAD2 transcript in Arabidopsis mRNA. PCR products from multiple plant lines from two different transformation events (different constructs) are shown. The larger band is the expected size for the DAD2 cDNA amplified with the oHWL29 and oPBG3 primers. B The smaller, unexpected band, was DNA sequenced. The sequencing trace data suggest there are two mis-spliced transcripts in the transformants when aligned to the DAD2 transgene sequence. Green = promoter, red = 10Myc tag, yellow = DAD2 CDS, orange = terminator, pink= original location of DAD2 intron, purple = predicted mis-spliced transcripts.
A
1 2 3 4 5 6 7
1 Ladder 1 kb+
2 proD14-10Myc-DAD2
3 proD14-10Myc-DAD2
4 proD14-10Myc-DAD2
5 proD14-DAD2
6 proD14-DAD2
7 NTC
B
Location of DAD2 intron
Predicted transcript 1
Predicted transcript 2
Sequencing trace

## Slide 7
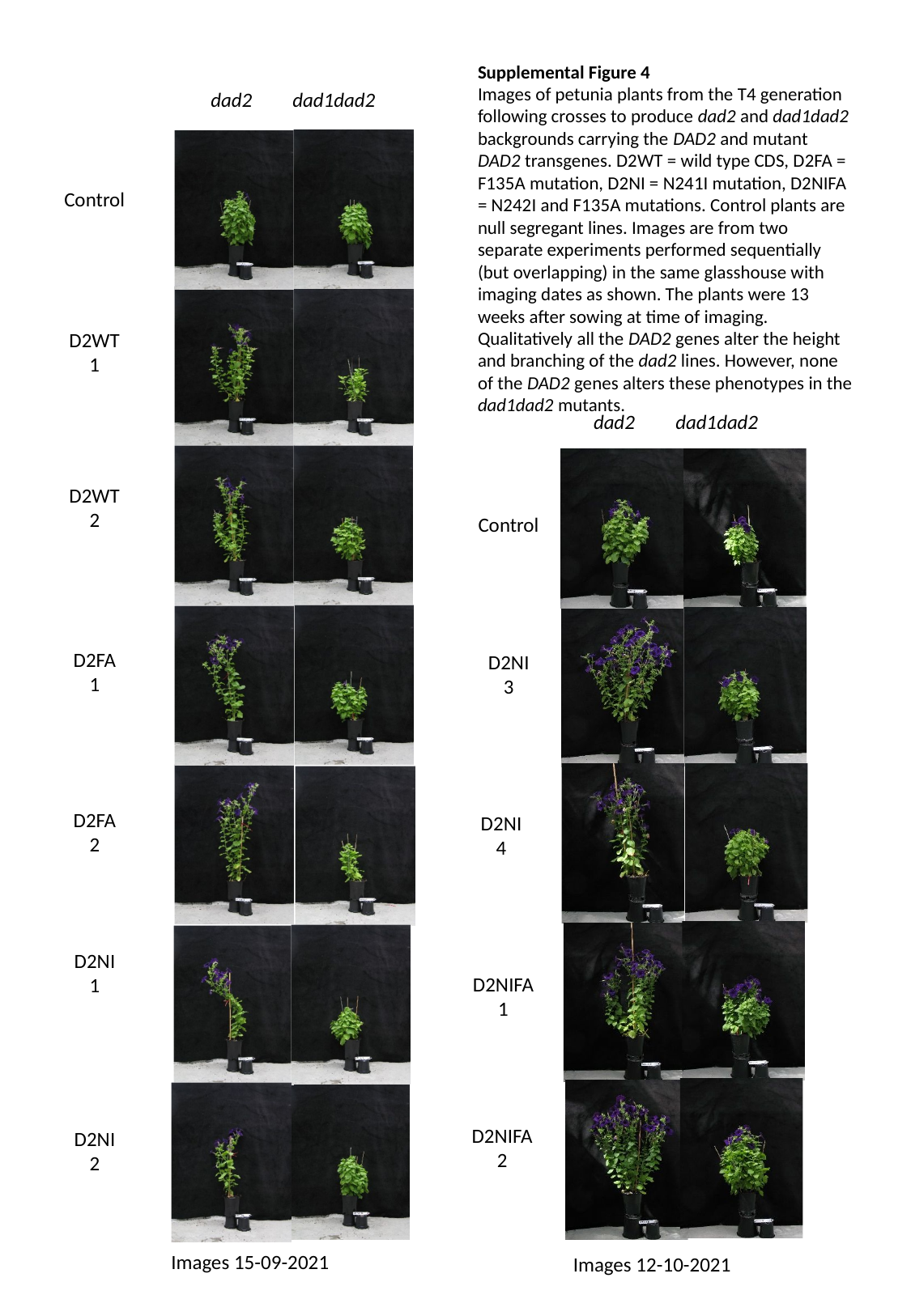

Supplemental Figure 4
Images of petunia plants from the T4 generation following crosses to produce dad2 and dad1dad2 backgrounds carrying the DAD2 and mutant DAD2 transgenes. D2WT = wild type CDS, D2FA = F135A mutation, D2NI = N241I mutation, D2NIFA = N242I and F135A mutations. Control plants are null segregant lines. Images are from two separate experiments performed sequentially (but overlapping) in the same glasshouse with imaging dates as shown. The plants were 13 weeks after sowing at time of imaging. Qualitatively all the DAD2 genes alter the height and branching of the dad2 lines. However, none of the DAD2 genes alters these phenotypes in the dad1dad2 mutants.
dad2
dad1dad2
Control
D2WT
1
D2WT
2
D2FA
1
D2FA
2
D2NI
1
D2NI
2
Images 15-09-2021
dad2
dad1dad2
Control
D2NI
3
D2NI4
D2NIFA
1
D2NIFA
2
Images 12-10-2021

## Slide 8
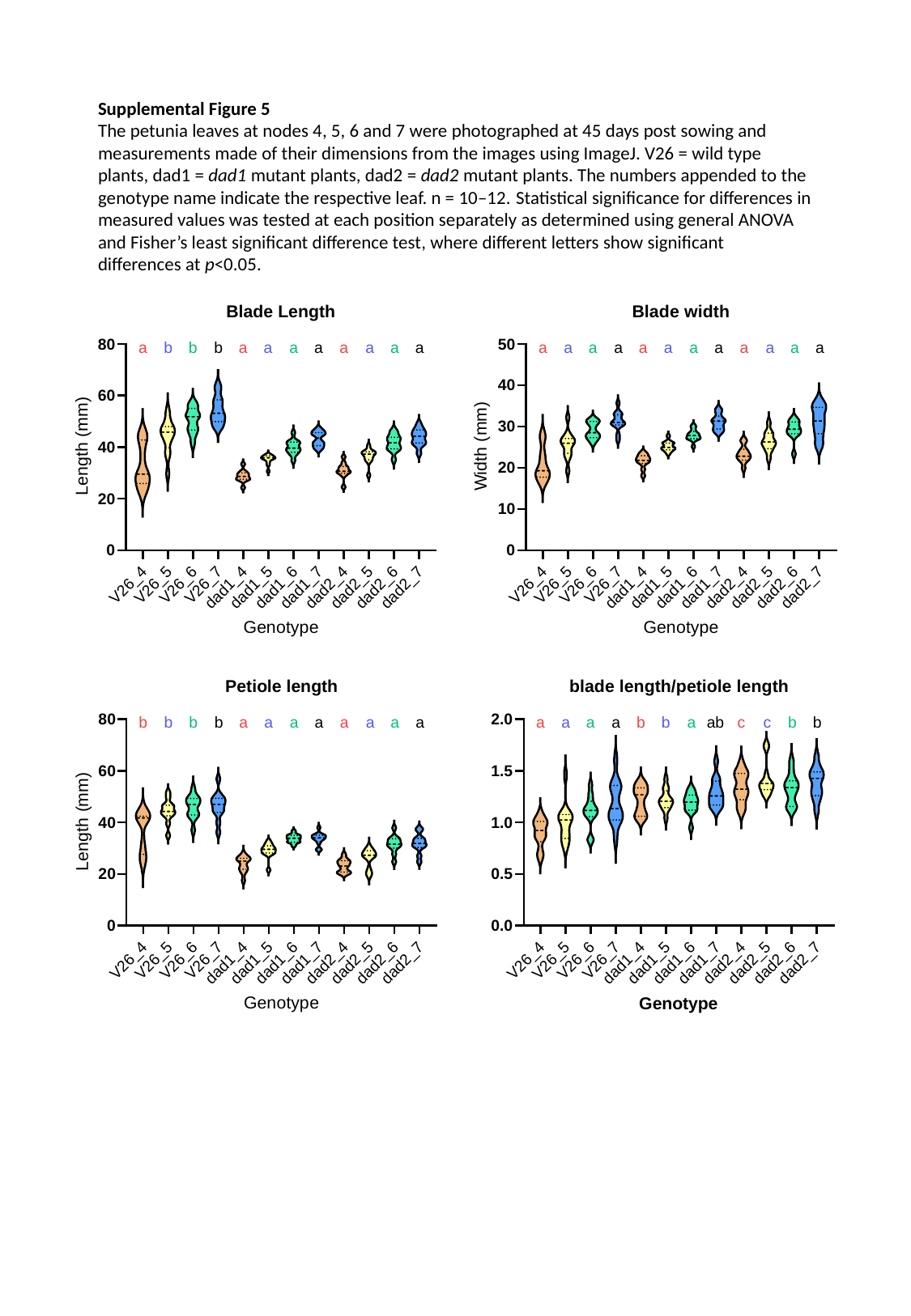

Supplemental Figure 5
The petunia leaves at nodes 4, 5, 6 and 7 were photographed at 45 days post sowing and measurements made of their dimensions from the images using ImageJ. V26 = wild type plants, dad1 = dad1 mutant plants, dad2 = dad2 mutant plants. The numbers appended to the genotype name indicate the respective leaf. n = 10–12. Statistical significance for differences in measured values was tested at each position separately as determined using general ANOVA and Fisher’s least significant difference test, where different letters show significant differences at p<0.05.

## Slide 9
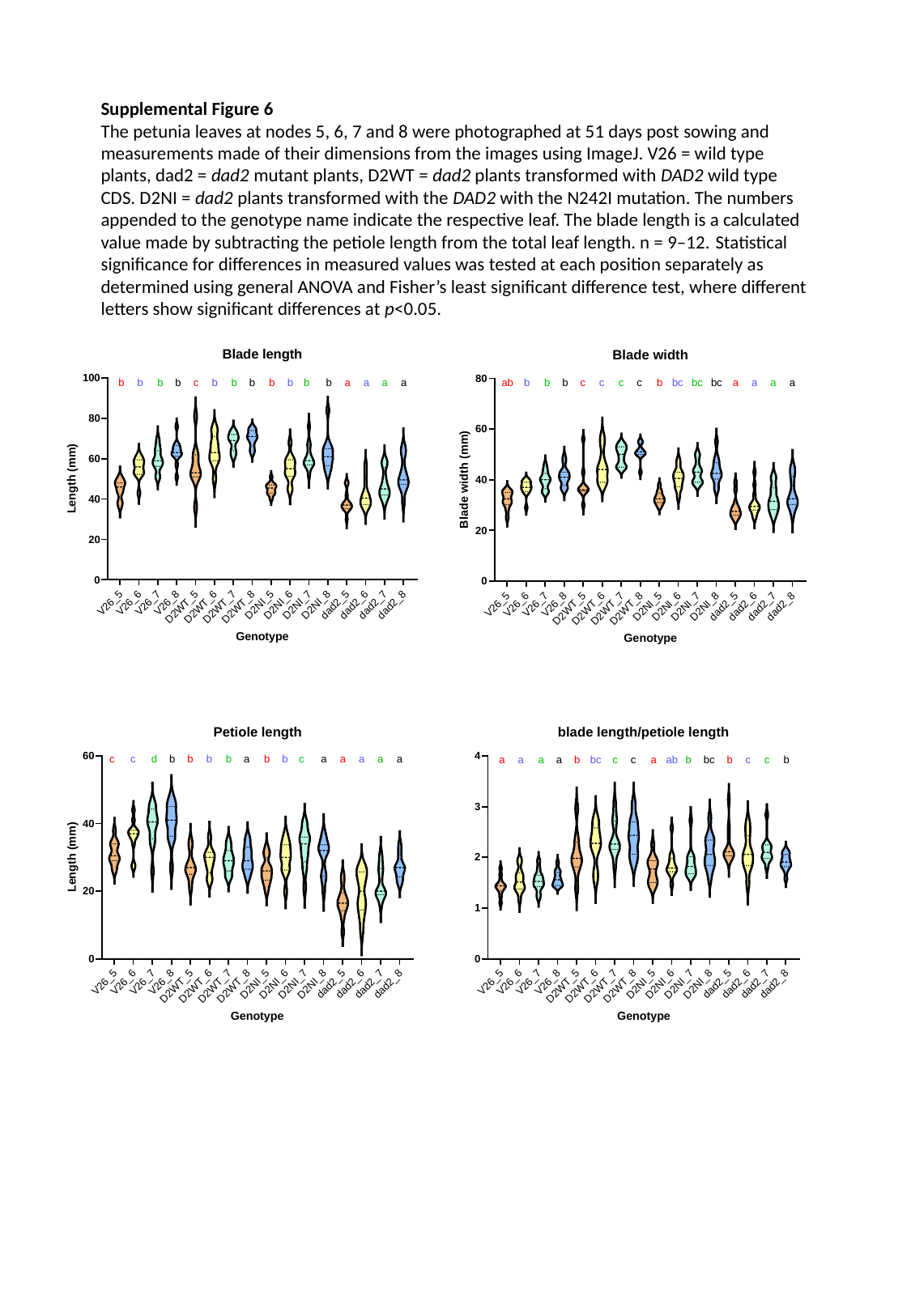

Supplemental Figure 6
The petunia leaves at nodes 5, 6, 7 and 8 were photographed at 51 days post sowing and measurements made of their dimensions from the images using ImageJ. V26 = wild type plants, dad2 = dad2 mutant plants, D2WT = dad2 plants transformed with DAD2 wild type CDS. D2NI = dad2 plants transformed with the DAD2 with the N242I mutation. The numbers appended to the genotype name indicate the respective leaf. The blade length is a calculated value made by subtracting the petiole length from the total leaf length. n = 9–12. Statistical significance for differences in measured values was tested at each position separately as determined using general ANOVA and Fisher’s least significant difference test, where different letters show significant differences at p<0.05.
